# Chicken Immune Response after *In Ovo* Immunization with Chimeric TLR5 Activating Flagellin of *Campylobacter jejuni*

**DOI:** 10.1371/journal.pone.0164837

**Published:** 2016-10-19

**Authors:** Katarzyna A. Radomska, Mahdi M. Vaezirad, Koen M. Verstappen, Marc M. S. M. Wösten, Jaap A. Wagenaar, Jos P. M. van Putten

**Affiliations:** 1 Department of Infectious Diseases and Immunology, Utrecht University, Utrecht, The Netherlands; 2 Central Veterinary Institute of Wageningen UR, Lelystad, The Netherlands; Sun Yat-Sen University, CHINA

## Abstract

*Campylobacter jejuni* is the main cause of bacterial food-borne diseases in developed countries. Chickens are the most important source of human infection. Vaccination of poultry is an attractive strategy to reduce the number of *C*. *jejuni* in the intestinal tract of chickens. We investigated the immunogenicity and protective efficacy of a recombinant *C*. *jejuni* flagellin-based subunit vaccine with intrinsic adjuvant activity. Toll-like receptor activation assays demonstrated the purity and TLR5 stimulating (adjuvant) activity of the vaccine. The antigen (20–40 μg) was administered *in ovo* to 18 day-old chicken embryos. Serum samples and intestinal content were assessed for antigen-specific systemic and mucosal humoral immune responses. *In ovo* vaccination resulted in the successful generation of IgY and IgM serum antibodies against the flagellin-based subunit vaccine as determined by ELISA and Western blotting. Vaccination did not induce significant amounts of flagellin-specific secretory IgA in the chicken intestine. Challenge of chickens with *C*. *jejuni* yielded similar intestinal colonization levels for vaccinated and control animals. Our results indicate that *in ovo* delivery of recombinant *C*. *jejuni* flagellin subunit vaccine is a feasible approach to yield a systemic humoral immune response in chickens but that a mucosal immune response may be needed to reduce *C*. *jejuni* colonization.

## Introduction

Campylobacteriosis is the most frequent bacterial zoonosis with estimated 9 million human cases and an economic burden of around 2.4 billion EUR each year in the European Union alone [1–3]. The main etiologic agent of human campylobacteriosis is *Campylobacter jejuni* (*C*. *jejuni*). Symptomatic *C*. *jejuni* infection usually manifests as an enterocolitis with a watery or bloody diarrhea, mostly accompanied with fever and abdominal pain. Infection may be followed by serious sequelae like reactive arthritis and Guillain-Barré syndrome [4–7]. The majority of human infections can be attributed to the consumption of *C*. *jejuni*-contaminated poultry meat products [8]. Reduction of *Campylobacter* in the chicken reservoir is therefore considered an effective strategy to reduce the public health burden [5]. It is estimated that a 2-log reduction of *C*. *jejuni* on chicken carcasses is sufficient to reduce the incidence of human campylobacteriosis by 30% [9].

One of the potential strategies to reduce *C*. *jejuni* colonization in broiler chickens is vaccination [3]. *C*. *jejuni* flagellin, the major subunit of the bacterial flagellum is an attractive candidate vaccine antigen [10]. Bacterial flagellins are highly immunogenic antigens in chickens [11–13] and their immunostimulatory properties including the activation of chicken Toll-like receptor 5 (TLR5) [14] make them potent vaccine adjuvants. Natural flagellin-specific antibodies likely contribute to maternal immunity in chickens [8]. Yet, vaccination of chickens with flagellin-based vaccines has yielded variable success [15–18]. Intraperitoneal immunization of 16 day-old chickens with heat-killed *C*. *jejuni* enriched with native flagellin followed by a booster two weeks later resulted in a ∼1–2 log reduction in cecal colonization [15]. Similarly, a recombinant *Campylobacter* flagellin fused to *Escherichia coli* heat-labile toxin administered orally at 14 day of age lowered the number of colonized animals [16]. Administration of heat-killed *C*. *jejuni* cells or flagellin to 16 day-old chicken embryos with increased the levels of flagellin-specific IgY (IgG), IgM and IgA antibodies in chicken sera, the levels of sIgA in the bile and intestinal scrapings, and the numbers of immunoglobulin-containing cells in the spleen and intestine. Yet, *in ovo* immunization combined with an oral or intraperitoneal booster given 7 days after hatch did not confer protection upon *C*. *jejuni* challenge [18,19].

In attempt to achieve protection against *C*. *jejuni*, we decided to improve the immune response to *Campylobacter* flagellin by introducing intrinsic adjuvant activity to the protein. In its natural form *C*. *jejuni* flagellin is unable to activate TLR5 in contrast to the flagella subunits of most other bacterial species [20,21]. Engineering of a chimeric *C*. *jejuni* flagellin that has the TLR5 activating domains of *Salmonella enterica* serovar Enteritidis (*S*. Enteritidis) resulted in a recombinant NHC flagellin that is able to activate TLR5 and thus potentially has intrinsic adjuvant activity [22]. In the present study, we determined the immunogenicity and protective efficacy of the recombinant NHC flagellin after *in ovo* delivery. Our results indicate that *in ovo* immunization with the flagellin-based subunit vaccine is an effective way to generate a specific systemic antibody response against *C*. *jejuni* but that this strategy is still not sufficient to provide protection against *C*. *jejuni* challenge.

## Materials and Methods

### Ethics statement

Animal experiments were performed at the experimental facilities of the Faculty of Veterinary Medicine of Utrecht University with the approval of the Animal Experiments Committee (Dierexperimentencommissie Utrecht, DEC Utrecht; Approval numbers: 2012.II.11.161 and 2013.II.02.009).

### Bacterial strains, cell lines and growth conditions

*E*. *coli* BL21 (DE3) Star (Invitrogen) was grown at 37°C on Luria-Bertani (LB) plates or in LB broth (Biotrading) supplemented with 100 μg x ml^-1^ of ampicillin. *C*. *jejuni* strain 81116 [23] and *C*. *jejuni* 81116 Δ*flaAB* [22] were routinely grown on agar plates with 5% saponin-lysed horse blood or in heart infusion (HI) broth (Biotrading) at 37°C or 42°C under microaerobic conditions (10% CO_2_, 5% O_2_, 85% N_2_). The presence of *Campylobacter* in cloacal swabs was tested using CCDA (charcoal cefoperazone deoxycholate agar) plates containing *Campylobacter* blood free selective agar base (Oxoid) and CCDA selective supplement (Oxoid) according to the manufacturer’s instructions. HeLa57A cell line stably transfected with a NF-κB luciferase reporter construct [24], was generously provided by Dr. R. T. Hay (Institute of Biomolecular Sciences, University of St. Andrews, St. Andrews, Scotland, U.K.). HeLa57A cells were propagated in Dulbecco modified Eagle medium (DMEM, Invitrogen) supplemented with 5% fetal calf serum (FCS) at 37°C under 10% CO_2_.

### Purification of NHC flagellin

The construction of the expression plasmid encoding His-tagged NHC flagellin has been described previously [22]. In brief, *E*. *coli* BL21 (DE3) Star harbouring the expression vector was grown for 16 h at 37°C, diluted in 100 ml of LB broth (optical density at 550 nm (OD_550_): 0.05), and then grown at 30°C. At OD_550_ of 0.5, 1 mM of IPTG was added to induce protein expression. After 4 h bacteria were harvested (4,400 × *g*, 20 min, 4°C) and suspended in 20 mM Tris, 100 mM NaCl (pH 8.0) containing EDTA-free complete protease inhibitor cocktail (Roche Diagnostics). After disruption of the bacteria by sonication (8 pulses of 15 s each with 20 s hold on ice), the insoluble fraction was collected after centrifugation (4,400 × *g*, 20 min, 4°C) and suspended in 8 M urea, 20 mM Tris, 20 mM imidazole, 250 mM NaCl (pH 8.0). After 16 h of incubation (RT, end-over-end rotation), nickel-nitrilotriacetic acid-agarose beads (Qiagen) were added. After 2 h the mixture was loaded into an empty column and extensively washed with 8 M urea, 20 mM Tris, 20 mM imidazole, 250 mM NaCl (pH 8.0). His-tagged protein was eluted with 8 M urea, 20 mM Tris, 250 mM imidazole, 250 mM NaCl (pH 8.0). Collected fractions were analyzed by SDS-PAGE. Fractions containing NHC protein were pooled and dialyzed (24 h, 4°C) against 10 mM Tris (pH 9.0). NHC protein was stored at -20°C in 4 M urea, 10 mM Tris (pH 9.0). Purified protein was analyzed by SDS-PAGE in combination with PageBlue Protein Staining (Thermo Fisher Scientific) and by Western blotting using monoclonal antibody CF1 (specific to *C*. *jejuni* 81116 FlaA flagellin) [25]. Protein concentrations were determined using the BCA protein assay kit (Thermo Fisher Scientific).

### Transient transfection

Plasmids encoding chicken Toll-like receptors (chTLR4, chTLR5, chTLR21, chTLR2t2, chTLR16) and the adapter proteins (chMD2, hCD14) have been described previously [14,26–28]. HeLa57A cells were transfected with the following combinations of plasmids: 1) chTLR2t2, chTLR16, hCD14; 2) chTLR4, chMD2, hCD14; 3) chTLR5; 4) chTLR21. HeLa57A cells grown in 6-well tissue culture plates were transfected transiently with 2 μg of DNA per well, using FuGENE HD Transfection Reagent (Promega) at a lipid to DNA ratio of 3 to 1. Cells were incubated with the DNA FuGENE HD Transfection Reagent mixture for 24 h. Then transfected cells were trypsinized and distributed in a 48-well plate (0.5 ml of DMEM with 5% fetal calf serum per well) and cultured for 24 h until use.

### TLR activation assay

Transfected cells were stimulated with NHC flagellin (1 μg x ml^-1^) or the following TLR ligands as a positive control: 1) Pam_3_CSK_4_ (100 ng x ml^-1^); 2) LPS of *Neisseria meningitidis* (100 ng x ml^-1^); 3) FliC flagellin of *S*. Enteritidis (1 μg x ml^-1^); 4) ODN 2006 (500 nM). Pam_3_CSK_4_ and ODN 2006 were purchased from Invivogen. LPS from *N*. *meningitidis* was purified as described previously [27]. The expression and purification of His-tagged *S*. Enteritidis (strain 90-13-706) FliC flagellin had been described elsewhere [14]. After 5 h of stimulation, the cells were washed twice with Dulbecco phosphate-buffered saline (DPBS), lysed with reporter lysis buffer (Promega), and frozen at -80°C. Luciferase activity was measured in a luminometer (TD-20/20, Turner Designs) after mixing cell lysate with luciferase reagent (Promega). Results were expressed as fold increase of NF-κB-induced luciferase activity in stimulated cells compared with non-stimulated cells.

### Determination of *C*. *jejuni* challenge dose

Seventeen day-old eggs (Ross 308) were purchased from a local hatchery (Kuikenbroederij Van Hulst B.V., Veldhoven, the Netherlands) and kept in an egg incubator. After hatch, chickens were randomly divided into 6 groups (10 animals per group). The *Campylobacter*-free status of the animals was verified at day 17 post-hatch by culture of cloacal swabs on CCDA plates. On day 18, five groups of chickens were challenged with 10^2^ up to 10^6^ bacteria per chicken. Before inoculation, *C*. *jejuni* was grown in HI broth (microaerobically, at 37°C) and diluted in HI broth to obtain the intended inoculation doses. Chickens were orally inoculated using a 1 ml syringe with 0.5 ml of bacterial suspension per chicken. One group received 0.5 ml of HI broth (control group). At four days post-inoculation (22 days of age), the chickens were sacrificed by means of cervical dislocation and the cecal content was tested for the presence of *C*. *jejuni* using CCDA plates.

### *In ovo* vaccination study

Prior to *in ovo* immunization, embryonated eggs (18.5 days of age) were randomly divided into 3 groups (10 eggs per group). Prior to injection, each egg was candled and disinfected with 10% chlorine. The eggs were immunized with 40 μg NHC protein (group 1), 20 μg NHC protein (group 2) or buffer (group 3). All injections were performed manually using MonoJect needles (0.7 mm x 25 mm; 22 x 1B). Each egg was injected with a total volume of 100 μl. The volume of samples containing NHC flagellin was adjusted to 100 μl with 10 mM Tris (pH 9.0), 20% glycerol, 5 mM sucrose, resulting in a final urea concentration of 0.4 M. The control group (group 3) was injected with the same solution (0.4 M urea, 10 mM Tris pH 9.0, 20% glycerol, 5 mM sucrose) but without NHC flagellin. To determine the humoral response induced by the immunization, blood samples were collected at days 12, 18 and 25 post-hatch. After blood clotting and centrifugation (2,000 x *g*, 5 min, 4°C), sera were collected and stored at -20°C until analyzed. At day 17 of age the *Campylobacter*-free status of the chicken was verified by culture of cloacal swabs on CCDA plates. On day 18, chickens were challenged with *C*. *jejuni* (10^5^ colony forming units per broiler). Animals were sacrificed at day 25 to collect intestinal scrapings for determination of antigen-specific secretory IgA (sIgA) and to determine the number of *C*. *jejuni* in the ceca by plate counting.

### Measurement of serum antibody levels

Antigen-specific antibody levels in sera were determined by enzyme-linked immunosorbent assay (ELISA). Flat-bottom 96-well plates (Corning) were coated (16 h) with NHC protein (2 μg/ well) in 0.05 M carbonate buffer, pH 9.6. Plates were rinsed five times with rinsing buffer (20 mM Tris, 150 mM NaCl, 0.05% Tween 20, pH 7.6) and blocked (2 h, 20°C) with rinsing buffer containing 5% FCS. After incubation (90 min) with serial dilutions of chicken sera in buffer with 1% FCS, the plates were rinsed, and incubated with 100 μl of goat anti-chicken IgY, IgM or IgA antibody conjugated to horseradish peroxidase (ABD Serotec) diluted: 1:2,000 in buffer with 1% FCS. After 1 h, plates were washed (5 times) and TMB substrate (BD OptEIA, BD Biosciences) was added. After 15 min the reaction was stopped by the addition of 1 M H_2_SO_4_. Absorbance was measured at 450 nm in a microplate reader (FLUOstar Omega, BMG Labtech). A standard reference serum (serum collected from 25-day old chicken challenged with *C*. *jejuni*) was included in all assays. The amount of antigen-specific IgY, IgM or IgA antibodies was expressed as a sample to reference ratio (S/R; relation of absorbance of tested serum sample to absorbance of the reference serum). Mean antibody titers were defined as the highest sera dilution giving statistically significant differences between the immunized and control group.

### Measurement of sIgA antibodies

The amount of antigen-specific sIgA antibodies in the (20-fold diluted) intestinal content was determined by ELISA, as described above. Antigen-specific sIgA levels were expressed in a relation to the total amount of sIgA per sample. Total amount of sIgA in the intestinal samples was determined using the Chicken IgA ELISA Quantitation Set (Bethyl Laboratories) according to the manufacturer’s instructions. Briefly, Nunc MaxiSorp flat-bottom 96-well plates were coated with unconjugated goat anti-chicken IgA (1 μg antibody/ well) and incubated at 20°C for 1 h. Plates were washed five times with buffer (50 mM Tris, 0.14 M NaCl, 0.05% Tween 20, pH 8.0) and blocked (30 min, 20°C) with the same buffer containing 1% BSA. Then, serial dilutions of intestinal scrapings in 1% BSA, 50 mM Tris, 0.14 M NaCl, 0.05% Tween 20, pH 8.0, were added to the wells. At the same time a range of dilutions of Chicken Reference Serum was prepared and added to the wells. After incubation (1 h, 20°C) and rinsing (5 times), goat anti-chicken IgA antibody conjugated with horseradish peroxidase (dilution: 1:75,000) was added. After 1 h of incubation (20°C) plates were washed (5 times) and the TMB substrate was added. After 15 min the reaction was stopped and the absorbance was measured.

### Western blot analysis of chicken sera

Purified flagellins and whole cell lysates of *C*. *jejuni* 81116 wild type and Δ*flaAB* were used in Western blot for analysis of the reactivity of chicken sera. His-tagged FlaA of *C*. *jejuni* strain 81116 was purified according to the procedure described above. Construction of the expression plasmid was described elsewhere [22]. Total cell lysates of *C*. *jejuni* 81116 wild type and Δ*flaAB* mutant were prepared from overnight cultures (5 ml HI, 16 h of incubation at 42°C, shaking at 180 rpm). Bacteria were harvested by centrifugation (4,400 × *g*, 20 min, 4°C), suspended in 1 ml of PBS, and sonicated (8 pulses of 15 s each with 20 s hold on ice). The flagellins (5 μg each) and total cell lysates of *C*. *jejuni* 81116 wild type and Δ*flaAB* mutant (10 μg each) were separated by 10% SDS-PAGE and electrotransferred onto a nitrocellulose membrane. The membrane was incubated (16 h, 4°C) with 5% skim milk in TBS-T (20 mM Tris, 150 mM NaCl, 0.05% Tween 20, pH 7.6) in order to block non-specific binding. Sera collected from 18 day-old chickens immunized with 40 μg of NHC protein were pooled and used as a probe (dilution 1:50 in TBS-T with 1% skim milk). Results were compared with the reactivity of pooled sera collected from the control group. After 1 h of incubation (20°C) the blots were washed with TBS-T and reactive bands were detected with goat anti-chicken IgY antibody conjugated with horseradish peroxidase (dilution: 1:2,000) and SuperSignal West Pico Chemiluminescent Substrate (Thermo Fisher Scientific).

### Statistical analysis

Statistical analysis was performed using one-way ANOVA with Bonferroni multiple comparison post test using GraphPad Prism software (version 6.01). Results were considered statistically significant at a level of α < 0.05.

## Results

### Analysis of purity and immunostimulatory properties of the vaccine antigen

**Fig 1:**
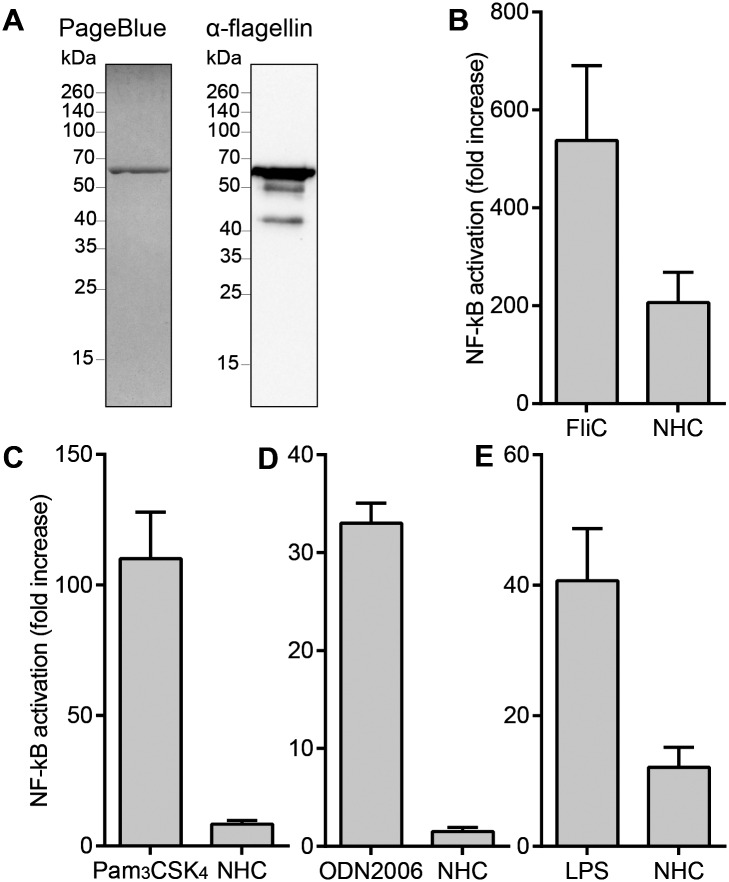
Quality properties of the NHC vaccine. (**A**) The NHC protein was analyzed by SDS-PAGE in combination with PageBlue Protein Staining and by Western blotting using a *C*. *jejuni* 81116 FlaA flagellin-specific antibody. (**B-E**) NF-κB luciferase activity of HeLa57A cells expressing (**B**) chTLR5, (**C**) chTLR2t2/chTLR16, (**D**) chTLR21 and (**E**) chTLR4, after stimulation (5 h) with NHC flagellin or the respective positive controls. Results are expressed as fold increase in stimulated cells versus non-stimulated cells and represent the mean ± SEM of three independent experiments.

In order to investigate whether chimeric NHC flagellin can be employed as a vaccine to reduce the *C*. *jejuni* load in chickens, the corresponding gene was expressed in *E*. *coli* and the His-tagged protein isolated by nickel-affinity chromatography. SDS-PAGE followed by PageBlue protein staining revealed that the isolated protein migrated as a single protein band with a molecular mass of approximately 63 kDa. Western blotting using the flagellin specific antibody CF1 as a probe showed reactivity with the major protein band as well with several minor bands that likely represent truncated flagellin fragments (Fig 1A).

The immune stimulatory activity of the purified NHC flagellin was determined in TLR activation assays. Hereto, HeLa57A cells stably transfected with the luciferase reporter gene under control of an NF-κB sensitive promoter and transiently transfected with the chicken *tlr5* gene were incubated with the purified NHC flagellin. Measurement of luciferase activity indicated potent stimulation of NF-κB for the chTLR5-expressing cells (Fig 1B), confirming the intrinsic immunostimulatory activity of the NHC protein. Stimulation of cells expressing chTLR2t2/chTLR16 or chTLR21 that respond to bacterial lipoproteins and DNA respectively, yielded no detectable NF-κB response, indicating that the isolated NHC protein was not contaminated with detectable amounts of lipopeptides or DNA (Fig 1C and 1D). NHC flagellin stimulation of cells expressing the chTLR4/MD2 complex that responds to LPS resulted in a small NF-κB response (Fig 1E). This contamination was considered acceptable in light of the high sensitivity of the assay and the MyD88-specific LPS response of chickens [27].

### Determination of challenge dose

**Fig 2:**
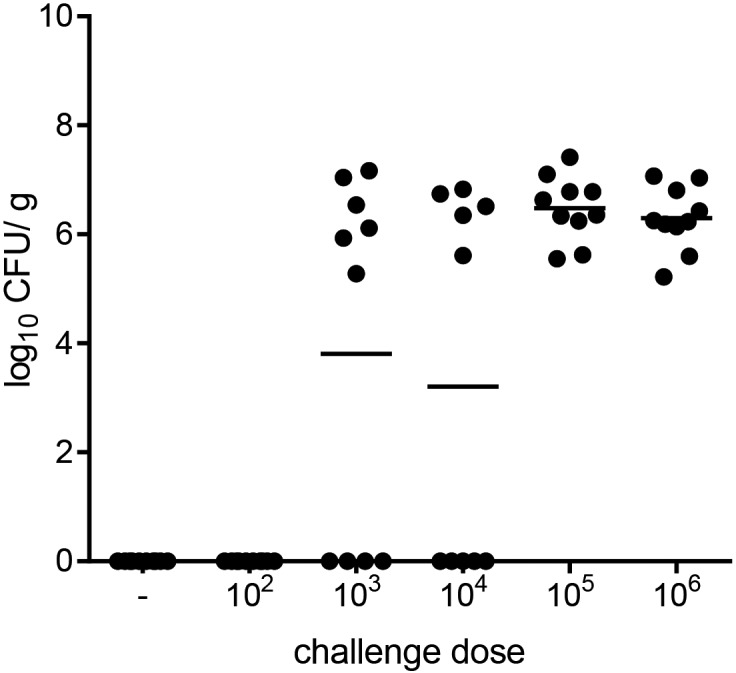
Determination of challenge dose of *C*. *jejuni* strain 81116. Chickens were orally inoculated with the indicated dose of *C*. *jejuni* strain 81116. Four days post-inoculation the number of *C*. *jejuni* in the chicken ceca was determined by plate counting and expressed as colony forming units per gram cecal content (CFU/ g). Symbols represent individual animal bacterial loads (log_10_ CFU/ g), and bars represent the averages.

As a first step to test the protection efficacy of the NHC flagellin vaccine against chicken colonization, we determined the optimal challenge dose of *C*. *jejuni* strain 81116. Hereto, different doses of *C*. *jejuni* (ranging from 10^2^–10^6^ bacteria per chicken) were orally inoculated to 18 day-old chickens to determine the lowest number of bacteria that provides colonization of all animals within the group. A dose equal to 10^5^ bacteria per broiler was needed to establish colonization of all chickens within the group (Fig 2). This dose was subsequently used in the *in ovo* vaccination study.

### Serum antibody production following *in ovo* vaccination with NHC flagellin

**Fig 3:**
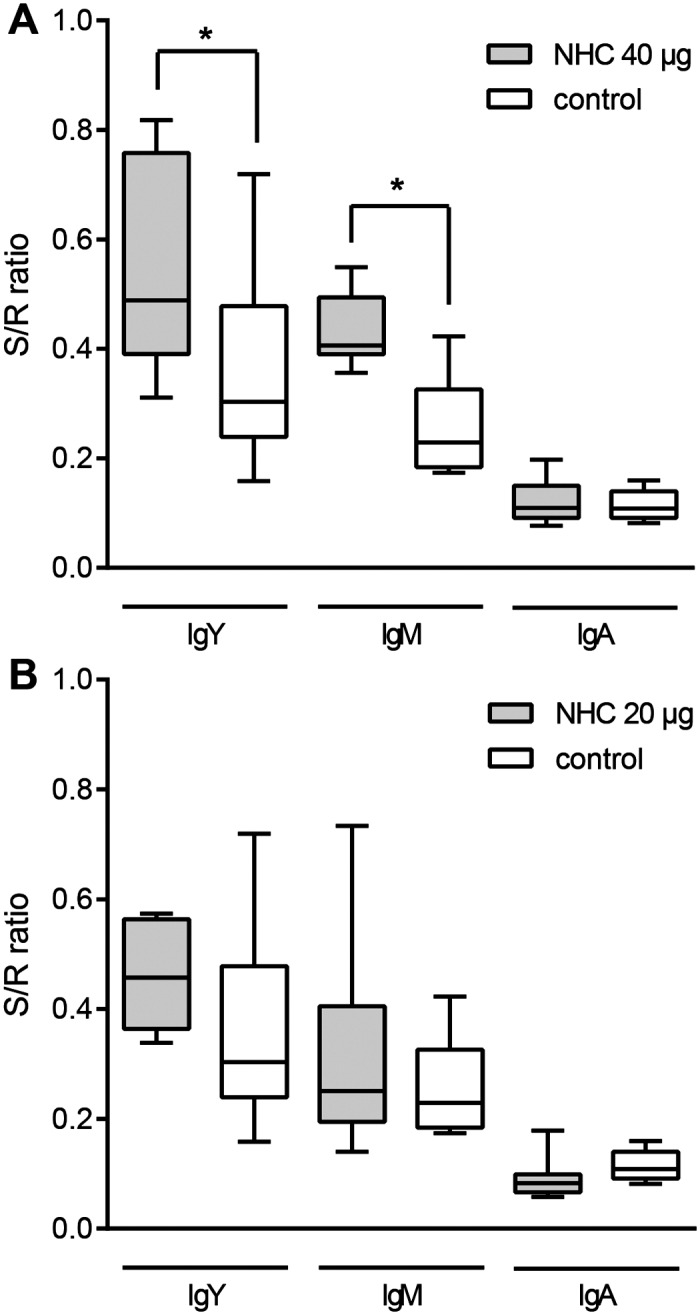
Antigen-specific serum antibody responses. NHC flagellin-specific antibody levels in the groups immunized with (**A**) 40 μg of NHC protein and (**B**) 20 μg of NHC protein, both compared to levels in the solvent injected control group. Antigen-specific IgY, IgM or IgA antibody levels were determined in 20-fold diluted sera collected from 12 day-old individual animals. Results are displayed as a box-and-whisker plot (S/R, sample to reference ratio). An asterisk indicates statistically significant differences (α < 0.05) between compared groups.

*In ovo* immunization was performed to determine whether NHC flagellin is immunogenic in chickens. NHC was administered to 18.5 day-old embryonated eggs at doses of 20 μg or 40 μg of protein. Injection of eggs with solvent served as negative control. The induction of antibodies was determined by ELISA using the NHC protein as antigen. Analysis of antibody levels in sera collected from individual animals at 12 days post-hatch revealed that *in ovo* vaccination with 40 μg of NHC resulted in NHC-specific IgY and IgM responses. Serum IgA levels were not different from the control group (Fig 3A). A vaccination dose of 20 μg of NHC protein was not sufficient to generate a systemic immune response, although IgY levels tended to be higher in the vaccinated animals (Fig 3B).

### Kinetics of the NHC-flagellin specific immune response

**Fig 4:**
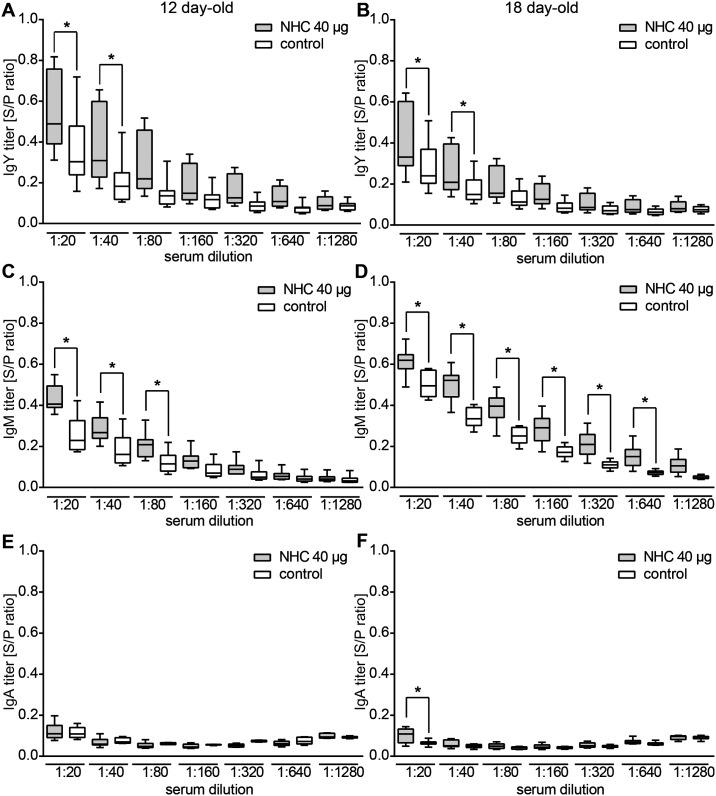
Kinetics of NHC-specific antibody levels following *in ovo* vaccination. ELISA results demonstrating the NHC flagellin-specific reactivity of serially diluted sera collected from (**A**, **C**, **E**) 12 day-old and (**B**, **D**, **F**) 18 day-old chickens injected with 40 μg of NHC flagellin or solvent (control). Serum IgY (**A**, **B**), IgM (**C**, **D**) and IgA (**E**, **F**) antibody responses are indicated. Graphs represent the analysis of individual animals, displayed as a box-and-whisker plot. The amount of antigen-specific antibodies was expressed in a relation to the reference sera (S/R, sample to reference ratio). An asterisk indicates statistically significant differences (α < 0.05) between groups.

To learn more about the titer and kinetics of the induced antibodies in the animals immunized with 40 μg of the NHC flagellin, we tested the antigen reactivity of serial dilutions of the sera of the vaccinated and control chickens collected at days 12 and 18 post-hatch. Significantly higher IgY levels in the vaccinated compared to control group were noted at serum dilutions of 1:20 and 1:40 both in the 12 and 18 day-old chickens (Fig 4A and 4B). Serum IgM antibody levels against the NHC flagellin appeared higher with significant differences compared to the control group ranging from 1:80 (12 day-old chickens) up to 1:640 (18 day-old chickens) (Fig 4C and 4D). Serum IgA levels were virtually similar between the vaccinated and control group in 12 and 18-day old chickens over the entire range of tested serum dilutions (Fig 4E and 4F), with the exception of a small difference at a serum dilution of 1:20 in 18 day-old chickens. In sera collected from group injected with 20 μg of NHC flagellin no differences in antigen-specific antibodies compared to the solvent-injected group were detected at any of three time points tested (data not shown).

### Chicken sera contain a high level of flagellin-specific antibodies

**Fig 5:**
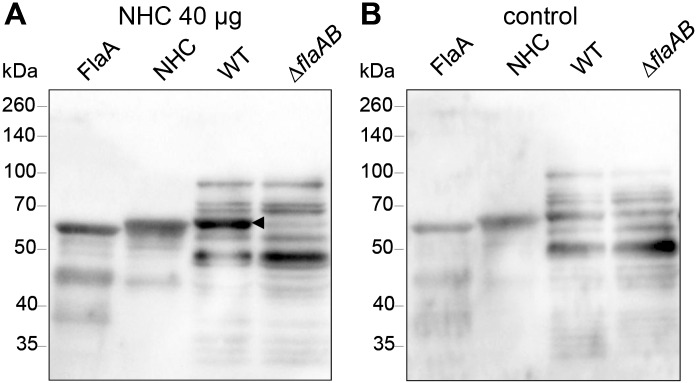
Western blot analysis of chicken sera. Purified flagellins (FlaA, NHC) and whole cell lysates of *C*. *jejuni* 81116 (wild type and Δ*flaAB*) were analyzed by Western blotting. Pooled sera collected from 18 day-old chickens injected with (**A**) NHC flagellin (40 μg) or (**B**) solvent were used as probes and detected with anti-chicken IgY antibody. Molecular mass is indicated in kilodaltons (kDa). The arrowhead indicates the reactivity of the sera with the native *C*. *jejuni* FlaA flagellin that is absent in the Δ*flaAB* mutant strain.

To verify the ELISA results, we tested the reactivity of the sera by Western blotting. Hereto, chimeric NHC protein and wild type *C*. *jejuni* FlaA flagellin were subjected to SDS-PAGE and transferred to nitrocellulose. Incubation of the blots with the pooled sera of from 18 day-old vaccinated and control animals showed strong reactivity of IgY antibodies of the vaccinated group against both wild type *C*. *jejuni* FlaA flagellin and the NHC (Fig 5A). Strikingly, the sera of the control group also reacted with these bands (Fig 5B). Further analysis of the reactivity of the sera using whole cell lysates of *C*. *jejuni* 81116 wild type as antigens demonstrated strong reactivity of the sera of both groups with multiple bands. Similar reactive bands were noted for strain 81116 Δ*flaAB* that lacks the *flaA* and *flaB* genes (Fig 5), indicating reactivity to non-flagellin antigens. The reactivity of chicken sera in the control group may also explain the relatively high IgY and IgM values measured for the control group in the ELISA (Fig 4A–4D). The antibody reactivity likely reflects the presence of maternally-derived IgY antibodies in chicken sera as the chickens were confirmed to be *Campylobacter*-free until day 18 (data not shown).

### *C*. *jejuni* challenge of vaccinated broilers and the effect on the mucosal immune response

**Fig 6:**
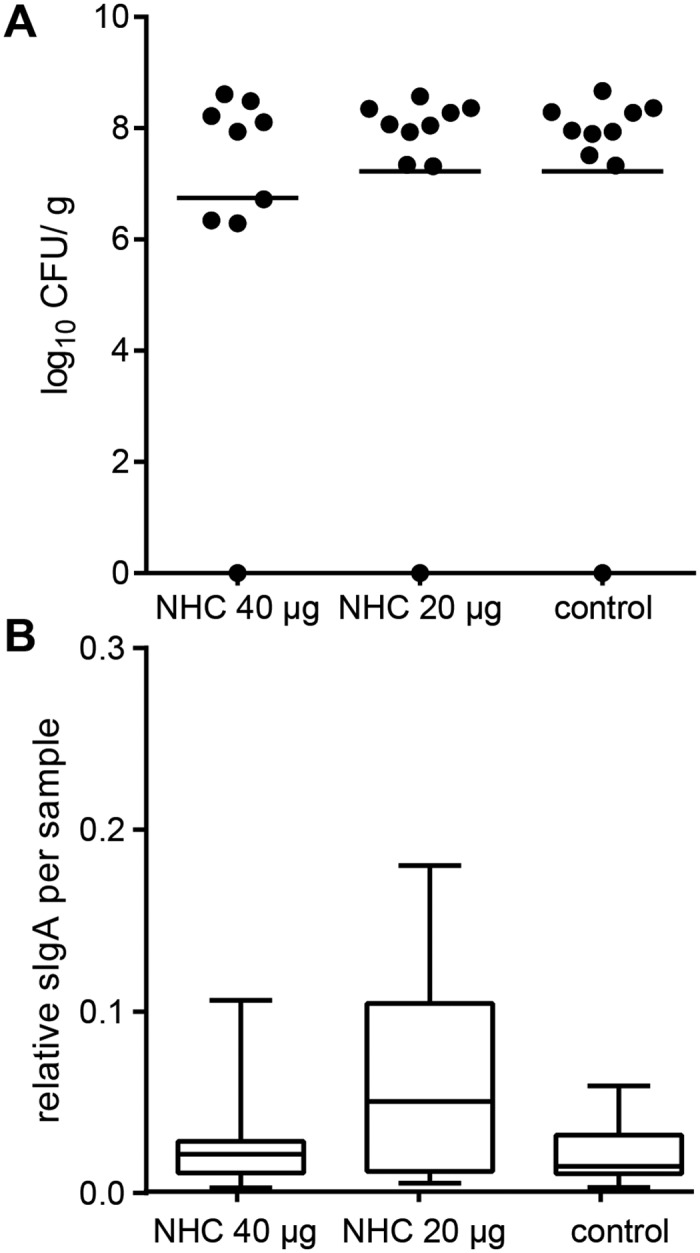
Cecal colonization of *C*. *jejuni* and sIgA responses in vaccinated and control chickens. (**A**) Chickens were challenged with *C*. *jejuni* at 18 day of age. One week post-inoculation (25 days of age) the number *C*. *jejuni* in the ceca was determined by plate counting (CFU/ g). Each symbol represents one chicken of the indicated groups, bars represent the mean number of log_10_ CFU/ g per group. (**B**) Antigen-specific sIgA in 20-times diluted cecal content from 25 day-old chickens (one week after *C*. *jejuni* challenge) as determined by ELISA. Graphs represent the analysis of individual animals of each of the indicated groups, displayed as a box-and-whisker plot.

Both the vaccinated and control group of chickens were challenged with *C*. *jejuni* strain 81116 at 18 days of age to verify whether *in ovo* vaccination conferred any protection. The number of *C*. *jejuni* in the ceca was determined one week after challenge by plate counting. The challenge resulted in intestinal *C*. *jejuni* colonization of almost all animals. The distribution of non-colonized chickens was equal among the vaccinated and control groups. In the vaccinated group (40 μg of NHC flagellin) slightly less *C*. *jejuni* were recovered from the ceca of three chickens (Fig 6A). This difference however, was not statistically significant.

In search for explanation for the lack of protection, we measured the levels of secretory IgA antibodies (sIgA) directed against the NHC flagellin in the intestines as these antibodies may prevent colonization of *Campylobacter* [12,29]. The levels of NHC flagellin-specific sIgA antibodies were determined for the intestinal contents of chickens sacrificed at one week after the *C*. *jejuni* challenge. No significant differences in the amount of antigen-specific sIgA were found among the vaccinated and the control groups (Fig 6B).

## Discussion

Our study demonstrates that *in ovo* immunization of embryonated chicken eggs with a chimeric *C*. *jejuni* flagellin-based subunit vaccine elicits antigen-specific IgY and IgM antibodies. *In ovo* vaccination with subunit vaccines is not common practice. Our successful immunization indicates that this approach is feasible. Previous work has shown that *in ovo* immunization with heat-killed *C*. *jejuni* elicits similar antibody responses as measured in sera collected from 5 day-old animals [18]. Whole cell vaccines (WCV) are attractive as they contain many antigens together with multiple TLR agonists (e.g. LPS, lipoproteins, DNA) that may act as natural adjuvants. The antigen composition of WCV however is often less well-defined and poorly controlled. The successful use of NHC flagellin subunit vaccine presented here indicates that a defined immunogenic bacterial antigen can be exploited as basis of a future vaccine as has been demonstrated for the *Eimeria* parasite [30]. As *C*. *jejuni* induces both systemic and secretory IgA antibody responses to a range of antigens including flagellin [31], incorporation of additional vaccine antigens may be also considered. Our subunit vaccine consisted of a chimeric *C*. *jejuni* flagellin that combined *C*. *jejuni* flagellin antigenicity with *S*. Enteritidis TLR5-immunostimulatory activity. This approach was chosen as *C*. *jejuni* flagellins lack endogenous TLR5 stimulating activity [22]. Only a small region of FlaA was replaced with TLR5 binding site of *S*. Enteritidis flagellin. In *S*. Enteritidis FliC this region is embedded within flagellar filament and it is not surface exposed [32]. The incorporation of the TLR5 binding region from *S*. Enteritidis FliC into *C*. *jejuni* FlaA flagellin resulted in a chimeric NHC protein that activates TLR5 and thus may have intrinsic adjuvant activity. The presence of antigen and adjuvant properties in a single molecule is considered to enhance the antigen presentation and the induction of an immune response [33]. The observed vaccine-dose dependent specific antibody responses indicate that at least 40 μg of NHC protein is needed to generate a significant response that lasts for weeks after hatch. For long-term protection a booster post-hatch may be considered. Alternatively, *C*. *jejuni* encountered in the broiler house may serve as “a natural booster”. In our study, bacterial challenge of the animals was indeed followed by a strong increase in antibody levels at day 25 post-hatch (data not shown). The long-term effect of a protective immune response on the bacterial colonization dynamics is difficult to predict and awaits the development of a protective vaccine.

Strikingly, the immunoglobulin isotype responses elicited by the vaccine showed different kinetics. The titer of NHC-specific IgM antibodies in sera collected from 18 day-old chickens was higher comparing to the analyzed sera from 12 day-old animals, whereas in the same time frame NHC flagellin-specific IgY levels remained constant or went slightly down. The IgA response was minimal and significantly elevated only at day 18. In the analysis of the chicken antibody kinetics it is important to consider the influence of maternal immunity, animal growth, and the duration of the antibody production in response to the vaccine. During the late stage of embryonic development the mother hen transfers mainly IgY antibodies from the yolk sac to the fetal circulation of the chicken, whereas IgM and IgA, which are predominantly present in the egg white, are ingested together with the egg white and transferred to the embryonic [34,35]. These antibodies provide (maternal) immunity during the first weeks of life, but gradually wane over time [8], resulting in a progressive reduction of overall IgY antibody titers. At the same time, the chicken grows fast in the first few weeks of life, resulting in strong dilution of the maternal antibodies in the chicken body. Combined, these factors result in a decrease of flagellin-specific IgY antibodies provided by the mother hen. Our ELISA and Western blot results indicate low levels of maternal IgY antibodies that cross-react with NHC flagellin in non-vaccinated chickens. In addition, to be effective, the vaccine-induced antibody production needs to keep pace with the chicken growth to prevent a further decrease in antibody titer. The gradual decrease in NHC flagellin-specific IgY levels up to day 18 suggests that the induced vaccine antibody synthesis may not be sufficient to provide a long lasting immunity. The increase in NHC flagellin-IgM reactivity over time, which was also noted for the non-vaccinated group, may be caused by the induction of cross-reactive antibodies by the natural microbiota.

Despite the induction of NHC flagellin-specific antibodies, the subunit vaccine did not prevent colonization of the intestinal tract upon challenge with the homologous strain of *C*. *jejuni*. This result is consistent with a previous study using heat-killed *C*. *jejuni* and flagellin protein as vaccines. *In ovo* immunization with these vaccines combined with an oral or intraperitoneal booster given at day 7 of age did not confer protection upon subsequent *C*. *jejuni* challenge [19]. One potential reason for the absence of protection is the lack of a potent mucosal sIgA response (Fig 6B). Secretory IgA, the major immunoglobulin isotype in the mucosa, is implicated into the clearance of *C*. *jejuni* from chicken intestine [12,29]. To steer the immune response selectively towards mucosal sIgA, a different route of antigen administration and the use of delivery vehicles can be considered [36]. An alternative explanation for the lack of protection may be that the generated antibodies directed against the recombinant protein did not react with exposed epitopes on native *C*. *jejuni* flagellin. *C*. *jejuni* flagellin is heavily decorated with sugars in contrast to the purified *E*. *coli* derived vaccine antigen [21,37]. It can be imagined that these carbohydrates prevent binding of the induced antibodies to the native *C*. *jejuni* flagella. This scenario is supported by the poor reactivity of flagellin-induced antibodies [38] and NHC flagellin-specific antibodies when intact *C*. *jejuni* were used as antigen in the ELISA (data not shown). Testing of the epitope shielding hypothesis awaits the synthesis of sufficient amounts of glycosylated NHC flagellin for use in vaccine studies.

In conclusion, our study provides evidence that *in ovo* immunization with *C*. *jejuni* flagellin-based subunit vaccine with intrinsic adjuvant activity induces specific antibodies in the chicken sera, which indicates that this approach is feasible. However, induction of potent mucosal immune response and/or the use of glycosylated flagellin as vaccine antigen may be needed to reduce bacterial colonization.
